# Dental Periostitis-Treatment

**Published:** 1862-02

**Authors:** J. Taft


### DENTAL PERIOSTITIS—TREATMENT.

BY J. TAFT.

Read before the Indiana State Dental Association, Jan. 15th, 1862.

UPON the pathology of this affection it is perhaps unnecessary to say anything; for, doubtless, you are all familiar with it. It is a subject, however, which should receive our most scrutinizing investigation. It has as many phases as there are different circumstances under which it may arise. All the conditions must be taken into consideration, in order to employ the most efficient treatment.

Inflammation of the dental periostium is of very frequent occurrence; it usually occurs with those teeth, the pulps of which are dead; having lost their internal vital supply, greater demand is made upon the external medium, and source of supply, and with this comes an increased susceptibility to irritation and inflammation.

In some cases a constitutional derangement will be quite sufficient to develop periostitis; in other cases, some local cause in connection with the general must operate before the condition will arise. Again, local causes alone will, in many cases, be sufficient to establish this condition. The treatment will, of course, be governed by these circumstances. It is then important to know the influences, and their character too, that operate in any given case. If the difficulty arises wholly from causes operating through the system, then the question arises, shall the treatment be general, local, or both combined? An inflammatory diathesis is the systemic condition that most frequently operates to produce dental periostitis. Under such a condition an acute form of the disease will supervene.

A chronic form of periostitis is often found with, and influenced by, a low grade of systemic vitality; in such cases, however, there are usually found, to some extent, local causes. An acute form of the disease, or that arising under the influence of an inflammatory diathesis, will always require general treatment. This will sometimes be quite sufficient to meet the whole difficulty. Treatment should be such as would tend to allay and counteract the inflammatory condition. In what this inflammatory condition consists, we do not now propose to consider.

There is often accompanying this condition a want of equilibrium of the circulation; and indeed this oftentimes is a source of much difficulty, when there is but little general phlogistic tendency; this will be exhibited by a full flow of blood to, and an increased amount of heat in the head, and cold extremities. A restoration of this equilibrium is always an important item, and sometimes will accomplish the desired object. This equilibrium is established by warm bathing, and friction of the extremities; and cold to the head, though the latter need not always be employed. An equilibrium will, in some cases, be much assisted by the administration of some saline purgative, of which Seidletz powders, Rochelle or Epsom salts, may be mentioned.

When a general phlogistic condition exists, the treatment must depend, of course, upon its character; if it is dependent upon a vitiated condition of the blood, then the case should pass into the hands of a physician; but if it is dependent upon some more ephemeral and less grave cause, then the wisdom of the dentist should enable him to meet the difficulty. In such cases the treatment should be an extension of that suggested for restoring the equilibrium of circulation. In a great many instances, this treatment is all that will be required for reducing dental periostitis, especially in cases of a recent origin.

In considering this subject, the importance of a thorough acquaintance with the subject of inflammation and its treatment is most apparent.

In most instances, local, in connection with general, treatment is required, though very many cases can be controlled by local treatment alone. The nature of the local treatment will depend upon the circumstances to which reference has already been made, in connection with other circumstances that attend it, such as the rapidity of its progress, whether it is limited in extent, or diffused, whether it possesses a high or low grade of vitality. To consider fully, however, the treatment for all the different forms of periostitis would far exceed the bounds within which we are limited. We propose here only to present that treatment which we think best adapted to the more common and obvious forms of dental periostitis.

In no case should we overlook or neglect the conditions referred to. In local treatment, the first indication is, to remove, so far as possible, any local cause of irritation or disease; after which, the treatment may be stimulant, by which a free and active circulation shall be induced, and in this way relieve the sluggish and unnatural circulation in the distended vessels, and by this means give them an opportunity to recover their normal tone. With the method of producing counter irritation you are doubtless all familiar, but we may mention the following, scarification; this should be made in the vicinity of the affected part. No application is, perhaps, as a counter irritant, more active than a mixture of chloroform, sulphuric ether and aquæ ammonia, equal parts, applied upon blotting paper to the affected part.

The absorbents should be stimulated to increased action. These play a very important part, almost everywhere, in the restoration of a diseased part to health. Constitutional treatment may be resorted to for the accomplishment of this object; in the treatment of dental periostitis, however, as a general thing, we do not think it advisable to resort to this, but think it far preferable to employ only local applications for this purpose. There is, perhaps, no agent better adapted than iodine, in the form of tincture, applied to the affected part with a camel's hair pencil; this is, perhaps, applicable in a greater variety of cases than any other agent. Depletion is also a valuable remedial resort, in this affection; there are various methods of accomplishing this, one of which is by the leech. This is the most effectual method of depletion in this treatment, and it can also be made most extensive, with the least injury to the parts. By this treatment, periostitis may be so far reduced, that pain and soreness will be at once removed, and the part afforded an opportunity for healthy action. Depletion by this means, in connection with the application of iodine, constitutes the local treatment for the great majority of cases of periostitis. Very seldom, if ever, will stimulants be called for, after the employment of the leech, and counter irritation. There are cases, however, in which depletion, neither by the leech or any other method, is indicated; for instance, where there is a low grade of vitality, and a poverty of blood, either in quantity or quality; in such cases, stimulants and tonics are indicated; the former locally, the latter through the system.

The most favorable time for the success of the treatment of periostitis is during its incipient stages. A very common fault, upon the part of the patient, is permitting the difficulty to continue for a considerable time without the proper attention to the first indications of the condition, which will usually be manifested by a slight soreness and protusion of the affected tooth from the socket.

Periostitis will sometimes be induced by some slight operation upon a dead tooth. This will occur when there is a strong systemic predisposition. Where such a predisposition exists, and an operation is immediately called for, the dentist would anticipate the difficulty, and employ such means as would prevent its occurrence. This treatment should be the same in kind, but not in degree, as that employed for the reduction of periostitis.

All this presupposes the ability of the operator to detect and to determine the character of pathological conditions, especially those from which these difficulties are liable to arise.

